# The Role of Neuroinflammation in Cellular Damage in Neurodegenerative Diseases

**DOI:** 10.1155/2020/9231452

**Published:** 2020-03-05

**Authors:** Sangu Muthuraju, Rahimah Zakaria, Mohan Kumar Muthu Karuppan, Badriya Al-Rahbi

**Affiliations:** ^1^Department of Neurosciences and Brain and Behaviour Cluster, School of Medical Sciences, Universiti Sains Malaysia, Jalan Raja Perempuan Zainab II, Kubang Kerian, 16150 Kota Bharu, Kelantan, Malaysia; ^2^Department of Pharmacological and Pharmaceutical Sciences, College of Pharmacy, University of Houston, Houston, 77240 TX, USA; ^3^Department of Physiology, School of Medical Sciences, Universiti Sains Malaysia, Jalan Raja Perempuan Zainab II, Kubang Kerian, 16150 Kota Bharu, Kelantan, Malaysia; ^4^Department of Immunology and Nano-Medicine, Herbert Wertheim College of Medicine, Florida International University, 11200 SW 8th St, Miami, FL 33199, USA; ^5^Oman College of Health Sciences, Muscat, Oman

The role of inflammatory mediators in the central nervous system (CNS) has been investigated in different types of neurodegenerative diseases, such as Alzheimer's disease (AD), Parkinson's disease (PD), and Huntington's disease (HD). Interestingly, these inflammatory mediators have a dual role in both proinflammatory and anti-inflammatory processes, upregulating and suppressing cellular damage in injury sites, respectively. Immediately upon injury or stress, the first and foremost responses are initiated by immune cells in the brain called microglia. Inflammation causes positive and negative symptoms in the periphery and CNS. In the CNS, inflammation is called neuroinflammation. Periphery inflammation could affect the CNS and induce neurological problems. For example, gut microbiota (GM) are present in the human intestine. The connection between GM and inflammation was found in AD. Recently, H. Shen et al. (2020) investigated inflammatory response activated by NLRP3 inflammasome which reaches the brain through circulation. This NLRP3 inflammasome could trigger microglial activation and form the pathological progression of AD. This article suggested that neuroinflammation could be triggered through periphery infections. Y. Wang et al. (2019) reported that neuroinflammation has the main role in the pathogenesis of AD, cerebral ischemia, and PD by CysLT1 and CysLT2 modulate inflammation during brain injury conditions. These are the possible targets for reducing inflammation.

Neuroinflammation is not only causing damage in the brain but is also driving or modulating the behavior of the human/animals. There are plentiful factors triggering neuroinflammation, especially stress, it is the major problem among adults promoting inflammation in the brain especially in the prefrontal cortex and motivate the animal to drink alcohol (HG Chuang et al. 2020). HG Chuang et al. reported that toll-like receptor 4 antagonist diminished alcohol seeking and drinking behavior of mice following restraint and social isolation stress. Besides, R Wang et al. (2020) demonstrated that maternal separation could enhance neuroinflammation in the hippocampus and prefrontal cortex due to depressive behavior. Stress at the early stage of life could induce short- and long-term depression through the production of cytokine and microglial activation.

In the line of research, the same group also studied that neuroinflammation in the striatum and cerebellum disturbed motor behavior following lipopolysaccharide (LPS) administration (data not published). This study directly helps to make new insight for approaching therapeutic for PD. I Parra et al. (2019) suggested that LPS administration is considered to be a better model for inflammatory and PD. ED Hamlett et al. (2020) reported that neuroinflammation induced memory loss in a down syndrome mouse model of Ts65Dn. Ts65Dn mice used for studying AD mouse model particularly age-related learning and memory loss associated with cholinergic neurons in the hippocampus and basal forebrain. ED Hamlett concluded that RvE1 could be potential therapy to reduce neuroinflammation and improve memory functions of persons with AD. This kind of preliminary findings lead us to establish animal studies to extend future research on AD and PD and further understanding of possible mechanisms to achieve effective therapy.

Recently, the literature has been reported that inflammation/neuroinflammation plays a key role in neurological diseases. Researchers have been involved to find out the crucial biomarkers of neuroinflammation in neurological diseases. Nevertheless, to understand neuroinflammation during disease condition is a major challenge for researchers. To find out, researchers and scientists, those who are working in the field of neuroinflammation and neurological diseases, making group, discuss the issues, these are the main idea for this special issue on “The role of neuroinflammation in cellular damage in neurodegenerative diseases”. Our special issue was appreciated and received many research and review articles and based on the reviewer's comments, some of the articles were accepted. The purpose of the special issue is almost achieved with the quality of the research articles published now. Last but not least, again in the near future, neuroinflammation-related neurological disease special issues would be posted soon.

